# Type IV Secretion and Signal Transduction of *Helicobacter pylori* CagA through Interactions with Host Cell Receptors

**DOI:** 10.3390/toxins9040115

**Published:** 2017-03-24

**Authors:** Steffen Backert, Nicole Tegtmeyer

**Affiliations:** Division of Microbiology, Department of Biology, Friedrich Alexander University Erlangen-Nuremberg, Staudtstr. 5, D-91058 Erlangen, Germany; nicole.tegtmeyer@fau.de

**Keywords:** protein kinase Akt, glycogen synthase kinase 3beta GSK3β, phosphatidylinositol 3-kinase PI3K, portioning kinase Par1b, β-catenin, p120 catenin, cortactin, phosphatase Shp-2, Janus kinase, STAT3

## Abstract

*Helicobacter pylori* is a highly successful human bacterium, which is exceptionally equipped to persistently inhabit the human stomach. Colonization by this pathogen is associated with gastric disorders ranging from chronic gastritis and peptic ulcers to cancer. Highly virulent *H. pylori* strains express the well-established adhesins BabA/B, SabA, AlpA/B, OipA, and HopQ, and a type IV secretion system (T4SS) encoded by the *cag* pathogenicity island (PAI). The adhesins ascertain intimate bacterial contact to gastric epithelial cells, while the T4SS represents an extracellular pilus-like structure for the translocation of the effector protein CagA. Numerous T4SS components including CagI, CagL, CagY, and CagA have been shown to target the integrin-β_1_ receptor followed by translocation of CagA across the host cell membrane. The interaction of CagA with membrane-anchored phosphatidylserine and CagA-containing outer membrane vesicles may also play a role in the delivery process. Translocated CagA undergoes tyrosine phosphorylation in C-terminal EPIYA-repeat motifs by oncogenic Src and Abl kinases. CagA then interacts with an array of host signaling proteins followed by their activation or inactivation in phosphorylation-dependent and phosphorylation-independent fashions. We now count about 25 host cell binding partners of intracellular CagA, which represent the highest quantity of all currently known virulence-associated effector proteins in the microbial world. Here we review the research progress in characterizing interactions of CagA with multiple host cell receptors in the gastric epithelium, including integrin-β_1_, EGFR, c-Met, CD44, E-cadherin, and gp130. The contribution of these interactions to *H. pylori* colonization, signal transduction, and gastric pathogenesis is discussed.

## 1. Introduction

*Helicobacter pylori* colonizes the stomach in about 50% of the human world population and is associated with chronic, often asymptomatic gastritis in all infected people. Successful acquisition of *H. pylori* requires an age-linked gastric physiology of the host and strain-specific features [1,2]. Colonization of *H. pylori* commonly occurs early in childhood and is characterized by lifelong persistence. Depending on multiple criteria, more severe gastric diseases including peptic ulcer disease can develop in up to 10%–15% of the infected individuals [3,4,5]. The presence of *H. pylori* is often associated with a strong inflammatory response, but the bacteria adapted various strategies during evolution to avoid clearance by the host defense systems. Humans carried *H. pylori* over at least 100,000 years and bacterial genetic features were used as a marker for tracing complex demographic events in human prehistory [6]. Due to this long time of co-evolution with man, it has been hypothesized that the accommodation of *H. pylori* may be beneficial for its host [7,8]. However, in our modern societies *H. pylori* is responsible for a high burden of morbidity and mortality due to several malignancies including mucosa-associated lymphoid tissue (MALT) lymphoma and gastric adenocarcinoma [3,4,5]. Gastric cancer is the fifth most incident malignancy in the world, with about 952,000 new cases and 723,000 deaths that occurred in 2012 [9]. The clinical outcome of *H. pylori* infections is dependent on a highly complex scenario of host-pathogen interactions. Disease progression is determined by various parameters including the genetic predisposition of the host, the bacterial genotype, and environmental factors [3,4,5]. The molecular and cellular strategies acquired by *H. pylori* to undermine host defense mechanisms and cause disease are under powerful investigation in many laboratories worldwide.

**Figure 1:**
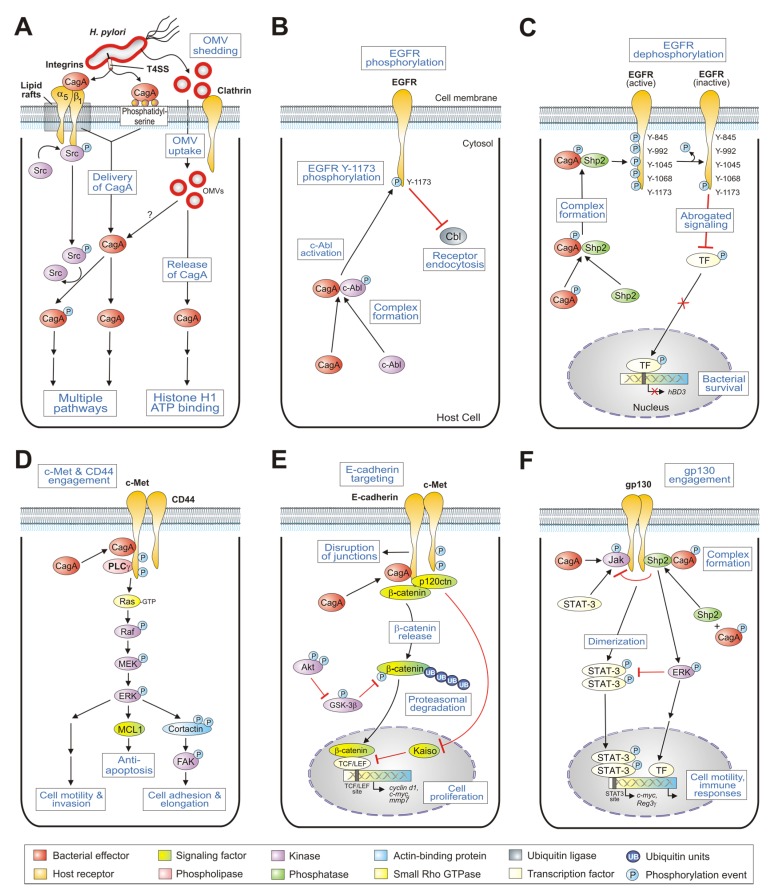
Schematic model for CagA-dependent interactions of *H. pylori* with host cell surface receptors and downstream signaling events. *H. pylori* delivers the effector protein CagA across both bacterial and host cell membranes into gastric epithelial cells. CagA targets various indicated host cell receptors in a type IV secretion-dependent (integrin-β_1_ or phosphatidylserine) or type IV secretion-independent (e.g., OMV uptake by clathrin-dependent or clathrin-independent mechanisms) manner to permit its translocation in host epithelial target cells (**A**). After delivery into the host cell cytoplasm, CagA can interact with the cytoplasmic tails of various protein receptors and trigger downstream signal transduction events including the inhibition of EGFR endocytosis (**B**); abrogation of EGFR-mediated hBD3 expression (**C**); enhancement of c-Met-mediated motogenic responses and anti-apoptosis (**D**); onset of cell proliferation through c-Met and catenins (**E**) and manipulation of gp130-mediated signaling (**F**). Black arrows highlight activated signal transduction pathways and red lines correspond to inhibitory cascades. The various signaling-engaged protein classes are highlighted with different colours as outlined at the bottom. For more details, see the text.

Dozens of bacterial virulence factors have been discovered, which are highly diverse both in their genetic polymorphisms and potential to induce pathogenicity. The *H. pylori* genomes contain more than 30 genes, which encode outer membrane proteins including several well-known adhesins such as BabA, SabA, AlpA/B, OipA, HopQ, and others which permit tight binding of the bacterium to host cell surface receptors [10,11,12,13,14]. Other established virulence-associated mechanisms include flagella-driven bacterial motility, urease-mediated neutralization of pH and inflammasome activation, VacA- and GGT-triggered immune suppression, protease HtrA-mediated cleavage of E-cadherin, and modification of host cell cholesterol [4,15,16,17,18,19]. In addition, the probably best studied *H. pylori* virulence determinant is the *cag*PAI-encoded T4SS and its effector protein CagA [20,21]. This secretion apparatus is unique compared to other members of two large T4SS subfamilies in the bacterial world, the conjugation systems, and the effector translocators [22]. Electron microscopy visualized the 41 nm T4SS core complex in the *H. pylori* membrane, composed of Cag3, CagM, CagT, CagX, and CagY proteins [23]. This core complex is connected to the extracellular T4SS pilus, produced upon host cell contact [24]. A number of T4SS proteins, including CagL, CagY, CagI, and CagA, are exposed at the pilus surface where they can interact with the integrin α_5_β_1_ host receptor followed by the translocation of CagA in epithelial cells [24,25,26,27,28]. Once this initial contact of the T4SS pilus is established, CagA appears at the pilus tip as indicated by immunogold labelling, suggesting that CagA could be transported through this appendage [24]. In addition, HopQ-mediated interaction with CEACAM receptors [29,30] and cholesterol in lipid rafts [31] have a function in CagA delivery, but their exact role is not yet clear. Upon translocation, CagA is sequentially phosphorylated (CagA^PY^) at EPIYA (Glu-Pro-Ile-Tyr-Ala) sequence repeats [32,33] by the concerted action of Src and Abl tyrosine kinases [34,35,36,37,38]. Translocated CagA then dysregulates the homeostatic signal transduction of gastric epithelial cells involved in chronic inflammation and malignancy by changing cell polarity, apoptosis, and proliferation [39,40,41]. Because of these cancer-promoting activities, CagA has been called the first bacterial oncoprotein [42]. Here we review our current knowledge on the multiple CagA functions with a focus on its interactions through host cell receptors in the gastric epithelium. The affected downstream signaling cascades and their importance in *H. pylori* pathogenesis are discussed. An overall model of the involved signal transduction pathways is shown in Figure 1.

## 2. Type IV Secretion-Dependent Delivery of CagA via Integrin-β_1_

Integrins are well-described mammalian cell adhesion receptors, which exhibit key functions in multiple processes determining normal and disease development [43]. Integrins consist of 24 heterodimeric family members comprising α and β chains, which facilitate the anchoring of cells to the extracellular matrix in healthy tissues [44]. It is now well established that *H. pylori* and its *cag* T4SS exploit integrin α_5_β_1_ as a receptor for CagA translocation [24,26,45,46]. After delivery, CagA localizes to the focal adhesions, where it hijacks host cell signaling pathways [24]. CagA of strain 26695 comprises 1,186 amino acids (aa), and is organized in several structural subdomains. Surface plasmon resonance experiments revealed that recombinant CagA can bind to purified integrin α_5_β_1_ with high affinity (dissociation constant *K_d_* = 0.15 nM) and the binding site has been mapped to the N-terminus of CagA [26,47].

The X-ray structures of several large N-terminal segments were reported, aa residues 1–884 [47], and residues 1–876 and 261–829 [48]. This roughly 100-kDa N-terminal subdomain of CagA revealed a unique combination of various protein folds. The core structure of this domain comprises a single-layer β-sheet, which is stabilized by two helical subdomains. This core is associated with a long helix, which creates a four-helix helical bundle. The conserved regions in the domain were mapped into four conserved surface-exposed patches (called CSP1–4), which were proposed to constitute sites for protein-protein interaction [47]. Yeast two-hybrid mapping and in vivo competition assays with recombinant CagA during *H. pylori* infection studies showed that the proximal part of the single-layer β-sheet, covering CSP4 (aa 303–404), is implicated in the interaction of CagA with integrin-β_1_ (Figure 1A). An important question therefore was why does CagA, as a translocated effector protein, bind to the extracellular domain of the integrin-β_1_ receptor with high affinity? Remarkably, the effective inhibition of CagA delivery by recombinant CagA fragments unambiguously shows that the interaction of CagA with integrin-β_1_ is a crucial prerequisite for CagA translocation into host cells [47]. This suggests a possible uptake mechanism of CagA together with its endocytosed receptor rather than a typical injection mechanism as known from effectors of type III secretion systems [49]. However, obtaining a more comprehensive picture of the complex docking by the T4SS pilus to the integrin-β_1_ receptor will be necessary in future studies, unraveling the concerted interaction properties of CagA and the other aforementioned T4SS factors (CagI, CagL, and CagY), which can also bind to integrin-β_1_ [24,26].

## 3. Internalization of CagA by Binding to Membrane-Associated Phosphatidylserine

Another model for T4SS-dependent CagA translocation has been proposed to include binding of pilus-exposed CagA to a host membrane phospholipid, phosphatidylserine [48,50]. There are several reported modules in proteins such as the pleckstrin homology (PH) domain, which can bind phospholipids [51]. Canonical PH domains exhibit a basic consensus motif (K-Xn-K/R-X-R) and a similar R-X-R sequence has been found in CagA of strain NCTC11637 comprising the arginine residues R-619 and R-621 [50]. Substitution of these arginines by alanines (CagA-R619/621A) abrogated the binding of phosphatidylserine by CagA in vitro. Infection of AGS cells with *H. pylori* carrying the same point mutations failed to translocate and phosphorylate CagA in gastric epithelial cells. These observations indicated that the interaction of CagA with phosphatidylserine is critically involved in the delivery of CagA into host cells (Figure 1A). Interestingly, sequence homology has been noted between the above phosphatidylserine-binding site in CagA and the lipid-binding Fes-CIP4 homology-Bin/Amphiphysin/Rvs (F-BAR) domains of mammalian proteins [52]. The uncovered similarities between a bacterial effector protein and eukaryotic F-BAR proteins suggest convergent evolution of CagA towards a similar function. However, it remains unclear why CagA cannot be delivered into various phosphatidylserine-expressing cell lines such as Hek293 [53], CHOK1, and GLC4 [54]. Thus, further experiments are required to study in more detail the process of phosphatidylserine-dependent delivery of CagA and its contribution to the above discussed integrin-mediated translocation pathway.

## 4. Type IV Secretion-Independent Uptake of CagA through Outer Membrane Vesicles

Outer membrane vesicles (OMVs) can be produced by virtually all Gram-negative bacteria and represent spherical buds of the outer membrane filled with periplasmic content [55]. OMVs are now recognized as a specific secretion vehicle, which provides a pathway to transport cargo (proteins, nucleic acids, lipids, and other small molecules) into the extracellular space, to other bacteria, as well as eukaryotic cells [56]. In this way, the production of OMVs allows bacteria to interact with their environment, and OMVs have been found to mediate diverse functions such as promoting pathogenesis, enabling bacterial survival during stress conditions, and regulating microbial interactions within bacterial communities. *H. pylori* also regularly buds-off OMVs, which were purified and characterized [57,58,59,60,61,62,63,64,65,66,67,68,69,70]. OMVs secreted by *H. pylori* have been detected not only during growth in vitro, but also in gastric biopsies and inside host cells [57,59]. *H. pylori* OMVs contain various lipids and at least 100 diverse proteins, including several described pathogenicity determinants such as adhesins, lipopolysaccharide, peptidoglycan, VacA, CagA, and others [57,59,66,67,68]. The population of OMVs is variable in size (20–300 nm) and differs both in protein and lipid content [68]. Thus, a scenario can be considered where different endocytic routes can be used for the delivery into gastric epithelial cells [67,71,72,73]. Indeed, the uptake of *H. pylori* OMVs is facilitated in microdomains by clathrin-dependent and clathrin-independent endocytic pathways (Figure 1A). The presence of CagA within the OMVs may represent an additional mechanism of delivery of this potent virulence factor into host cells compared to the internalization routes discussed above. The importance of OMVs carrying CagA was investigated in cell-to-cell junctions and the ATP-binding proteome of Caco-2 cells, using proteomics, mass spectrometry, and fluorescence imaging [74]. OMV-associated CagA was found to co-localize with the tight junction protein ZO-1 and induced histone H1 binding to ATP [74]. The distribution and position of ATP-H1 at particular cell chromosomes may have both positive and negative effects on host gene transcription. It will be interesting to investigate if CagA is phosphorylated upon OMV-mediated host cell internalization and whether the same or different signaling cascades are turned-on/-off compared to T4SS-translocated CagA. Taken together, the delivery of CagA by OMVs is an intriguing new strategy and may have an impact on the outcome of *H. pylori* infection, which needs to be studied in more detail in future experiments.

## 5. Inhibition of EGFR Endocytosis by the Non-Receptor Kinase c-Abl and CagA

Epidermal growth factor receptor (EGFR) belongs to the ErbB group of receptor tyrosine kinases (RTKs), and represents a key player in normal cell growth, survival, proliferation, and wound healing [75,76]. Ligand binding induces homo- and/or heterodimerization of EGFR in the membrane, leading to kinase activation by autophosphorylation of various tyrosine residues in the intracellular tail and subsequent onset of downstream signaling events. In contrast, dysregulation and mutation of ErbB RTKs can lead to oncogenic transformation and other pathologies [77,78]. Interestingly, several early studies have documented the occurrence of upregulated EGF and EGFR levels in *H. pylori*-infected gastric biopsies in vivo [79,80]. Further reports have shown that infection of gastric epithelial cells in vitro resulted in a rapid transactivation of EGFR via matrix metalloproteinase (MMP)-dependent signaling [81,82], regulating various responses including the activation of pro-inflammatory transcription factor NF-κB, cell spreading, anti-apoptosis, and suppression of the H,K-ATPase α inhibiting gastric acid secretion [81,82,83,84,85,86,87]. However, a crucial mechanism controlling EGFR activity in healthy epithelial cells is ligand-triggered endocytosis, where the internalized receptor recycles back to the plasma membrane or is targeted for degradation [88,89]. Remarkably, while the early *H. pylori*-mediated transactivation of EGFR is independent of CagA [81,82,83], translocated CagA was required for inhibiting EGFR endocytosis and subsequent degradation during prolonged infections [90]. Activated c-Abl kinase was also essential and induced the upregulation of EGFR surface expression in infected cells through the phosphorylation of EGFR at Y-1173. In addition, elevated EGFR surface exposure was observed in cells transfected with wild-type CagA, providing further evidence for CagA dependency [90]. However, expression of a phosphorylation-deficient CagA point mutant exhibited EGFR surface levels similar to cells expressing wild-type CagA, suggesting that CagA phosphorylation is not involved. Taken together, these findings suggest a novel CagA-dependent but phosphorylation-independent activation mechanism of the c-Abl kinase, which in turn phosphorylates EGFR at Y-1173 (Figure 1B). A critical regulator of ligand-dependent EGFR downregulation is ubiquitin ligase Cbl, which mediates ligand-induced ubiquitination and the subsequent degradation of EGFR [89]. Activated Abl allows EGFR to escape Cbl-dependent downregulation by preventing the recruitment of Cbl to the plasma membrane [88]. These data support a fascinating strategy of how *H. pylori* manipulates selective host receptor signaling by a novel CagA- and c-Abl-dependent endocytic mechanism.

## 6. CagA Abrogates Human β-Defensin 3 Expression by the Dephosphorylation of EGFR

Antimicrobial peptides such as the β-defensins are important weapons of the host innate immune system acting as a first barrier against infectious microbes [91]. *H. pylori* is able to persistently colonize its host despite inducing the expression of several antimicrobial peptides, including human β-defensin 3 (hBD3). hBD3 is highly active against *H. pylori* in vitro and is stimulated by EGFR signaling during early times of infection [92,93]. EGFR engagement induces signaling of the p38 mitogen-activated protein (MAP) kinase and Janus kinase/signal transducers and activators of transcription (JAK/STAT) to the nucleus and hBD3 transcription [92,93]. Interestingly, during prolonged infection times, hBD3 mRNA and protein expression was subsequently inhibited in a CagA-dependent manner [92]. Upon delivery and phosphorylation in host cells, CagA^PY^ binds to the SH2 domain of tyrosine phosphatase Shp2 [94]. This interaction activated the phosphatase activity of Shp2 towards various EGFR phosphorylation sites. In particular, phosphorylation at Y-845, Y-992, Y-1045, and Y-1068 in EGFR was significantly downregulated by Shp2 during prolonged infection (Figure 1C). These dephosphorylation events were found to stop EGFR activation and nuclear signaling to hBD3, and re-established *H. pylori* viability [92]. Interestingly, the phosphorylation intensity of the Y-1173 residue was not affected by CagA^PY^-Shp2 engagement. These findings led to a model in which the CagA^PY^-Shp2 signaling complex controls selected EGFR phosphorylation sites and inhibition of hBD3 release [92]. In agreement with these in vitro studies, hBD3 protein levels were significantly decreased in *H. pylori* infected vs. non-infected gastric biopsies [95]. Altogether, the above discussed experiments established a novel mechanism showing how translocation and phosphorylation of CagA inhibits the host innate immune response, which support the persistence of *H. pylori* infections.

## 7. CagA Protein Targets the c-Met Receptor and Enhances the Motogenic Host Cell Response

c-Met, also known as the receptor for hepatocyte growth factor (HGF), promotes tissue morphogenesis, wound healing, and organ homeostasis [96]. However, various mutations in the c-Met gene leading to overexpression of c-Met are implicated in late-stage cancer metastasis and poor patient prognosis. Early studies have shown that *H. pylori* infection triggers profound c-Met phosphorylation in AGS cells in a T4SS-dependent, but CagA-independent manner [97]. Co-immunoprecipitation experiments demonstrated that translocated CagA can interact with the intracellular domain of c-Met, which enhanced c-Met signaling and a forceful motogenic response of infected AGS cells. Furthermore, CagA associates with the phospholipase C-γ (PLCγ), but not with the well-known adapter protein Grb2-associated binder 1 (Gab-1) or growth factor receptor-bound protein 2 (Grb-2). The *H. pylori*-induced motogenic response is suppressed by the pharmacological inhibition of phospholipase C-γ and ERK1/2 MAP kinases (Figure 1D), but not by inhibition of phosphatidylinositol 3-kinase (PI3K) [97]. Profound activation of the MAP kinase pathway by translocated CagA has been confirmed by other groups [98], which can induce NF-κB [99] and the anti-apoptotic myeloid leukemia cell differentiation protein 1 (MCL1, Figure 1D) [85]. However, the downstream targets of ERK1/2 kinases to trigger cell motility during infection were unknown for a long time. A recent study has shown that *H. pylori* targets the ERK kinase to phosphorylate the actin-binding protein cortactin at the serine residues S-405 and S-418 [100]. Upon infection, serine-phosphorylated cortactin was found to interact with and stimulate the activity of focal adhesion kinase (FAK) which triggered enhanced cell adhesion and elongation (Figure 1D). It has been proposed that *H. pylori* targets cortactin to protect the gastric epithelium from excessive cell lifting and ensure sustained infection in the stomach.

## 8. CagA Associates with c-Met and CD44 Activating Host Cell Proliferation

Cluster of differentiation 44 (CD44) is a multifunctional transmembrane glycoprotein, which was initially described as a receptor of hyaluronan with important roles in normal physiological and pathological processes [101]. Extensive research has shown that high CD44 expression correlated with the phenotypes of cancer stem cells and the epithelial-mesenchymal transition (EMT), thereby contributing to tumor invasion and metastasis. Interestingly, CD44 also acts as a co-receptor for c-Met, which led to the proposal that it may play a functional role in *H. pylori*-associated pathogenicity. Currently, gastric organoids are developed as a popular experimental model system for *H. pylori* infection [102,103]. Infection of mouse- and human-derived gastric organoids with wild-type *H. pylori* induced epithelial cell proliferation that correlated with c-Met phosphorylation as analyzed by immunoprecipitation and Western blotting [104,105]. Remarkably, CagA and CD44 co-immunoprecipitated with phosphorylated c-Met [105]. The formation of this trimeric complex did not appear in organoids infected with an isogenic *cagA* mutant, suggesting that translocated CagA is triggering these protein-protein interactions (Figure 1D). In addition, organoids derived from CD44-deficient mouse stomachs were infected with *H. pylori* and epithelial cell proliferation was abrogated [105]. Moreover, human-derived fundic gastric organoids infected with wild-type *H. pylori* showed prominent cell proliferation, but this phenotype was blocked in the presence of the CD44-specific peptide inhibitor Pep1. In the well-established Mongolian gerbil infection model of gastric cancer, animals pre-treated with Pep1 resulted in the inhibition of *H. pylori*-induced proliferation and associated atrophic gastritis [105]. Taken together, these studies unravel an important functional role of c-Met and CD44 in *H. pylori*-induced epithelial cell proliferation correlating with gastric pathogenesis.

## 9. CagA Targets E-Cadherin and Deregulates Catenin Signaling

E-cadherin represents a calcium-dependent cell-to-cell adhesion receptor in epithelial cells, which controls epithelial morphology and differentiation in various tissues [106,107]. Transfection studies have indicated that non-phosphorylated CagA can physically interact with E-cadherin [108]. The intracellular domain of E-cadherin interacts with members of the catenin family, in particular β-catenin and p120-catenin (p120^ctn^). Detailed analysis using a series of CagAs from Western and East Asian strains revealed that deregulation of β-catenin requires the CagA CRPIA or multimerization sequence [109]. It appears that the CagA/E-cadherin complex impairs the interaction between E-cadherin and pro-oncogenic β-catenin, resulting in the cytoplasmic and nuclear accumulation of β-catenin (Figure 1E). Nuclear accumulation of β-catenin is also increased in biopsies from patients with early and late gastric carcinoma [110,111] and in the gastric epithelium of Mongolian gerbils infected with the highly cancerogenic *H. pylori* strain 7.13 [112]. In MKN28 and MKN45 human gastric epithelial cells, transfected CagA deregulated β-catenin and then transactivated T-cell factor/lymphoid enhancer factor (TCF/LEF) and CDX1 transcription factor to induce the expression of cancer-associated Wnt target genes, such as *c-myc* and *cyclin d1* [108]. A similar observation was reported in a study investigating p120^ctn^ function in this context. Upon infection, nuclear translocation of p120^ctn^ was detected, which relieved Kaiso-mediated transcriptional repression of the metalloproteinase MMP-7 (Figure 1E). This signaling required a functional T4SS, but was independent of CagA [113]. In addition, intracellular CagA transactivates p21(WAF1/Cip1) in a phosphorylation-independent manner. Consequently, CagA induces aberrant expression of an intestinal-differentiation marker, goblet-cell mucin MUC2, in gastric epithelial cells that have been arrested in G1 by p21 (WAF1/Cip1) [108]. Infection of MDCK cells led to the suppression of serine/threonine phosphorylation and ubiquitin-dependent degradation of β-catenin and to up-regulation of TCF/LEF-dependent transcription [114]. The impaired phosphorylation of β-catenin was accompanied by an increase of glycogen synthase kinase 3beta (GSK-3β) phosphorylation. In fact, Akt kinase (also called protein kinase B, PKB) directly phosphorylates and thereby inhibits GSK-3β, leading to impaired phosphorylation and stabilization of the β-catenin protein [111]. In agreement with these observations, pharmacological inhibition or siRNA knockdown of Akt kinase restored serine/threonine phosphorylation of β-catenin [114]. Taken together, these results indicate that perturbation of the E-cadherin/β-catenin complex as well as Akt and GSK-3β signaling induced by *H. pylori* CagA play important roles in host cell proliferation and the development of intestinal metaplasia and gastric cancer.

## 10. CagA Associates with c-Met, E-Cadherin, and p120^ctn^ with an Impact on Host Cell Invasion

Further studies of CagA signaling focused on the differences seen during *H. pylori* infection of non-polarized vs. polarized epithelial cell lines. The widely used non-polarized AGS cells do not form proper cell-to-cell junctions because they are E-cadherin deficient. Oliviera and co-workers found that *H. pylori* induces AGS cell invasion in collagen type-I and matrigel invasion assays [115]. Pharmacological inhibition or siRNA-mediated silencing of c-Met profoundly inhibited AGS cell invasion. Studies with different *H. pylori* mutants revealed that cell invasion, c-Met tyrosine phosphorylation, and elevated metalloproteinase MMP-2 and MMP-9 activity in AGS cells required the presence of a functional T4SS [115]. Further infection studies utilized the polarized E-cadherin-expressing cell line NCI-N87. Expression of E-cadherin was sufficient to suppress not only the *H. pylori*-mediated cell-invasive phenotype, but also c-Met and p120^ctn^ tyrosine phosphorylation [116]. *H. pylori* infection of NCI-N87 cells also led to increased interactions between c-Met and E-cadherin, c-Met and p120^ctn^, and E-cadherin and p120^ctn^ (Figure 1E). Infection assays of NCI-N87 cells further showed that *H. pylori* CagA forms a complex with c-Met, E-cadherin, and p120^ctn^. Moreover, using siRNA, it was demonstrated that interactions between CagA and E-cadherin and between CagA and p120^ctn^ were established through c-Met. Finally, the multiprotein complex was not detected in infected AGS cells, but could be restored in AGS that were stably transduced with E-cadherin. Interestingly, this complex abrogated tyrosine phosphorylation of c-Met and p120^ctn^, and suppressed the above-described cell-invasive phenotype induced by *H. pylori* [116]. The molecular basis for this observation is not yet fully clear. Future studies should investigate this signaling more thoroughly and also the possible crosstalk between the reported CagA activities and serine protease HtrA during infection, which was shown to cleave E-cadherin both in vitro and during infection [16,117].

## 11. CagA Determines gp130-Activated Shp2/ERK and JAK/STAT Signal Transduction

Activation of the glycoprotein 130 (gp130) receptor and associated downstream JAK/STAT signaling cascades have been shown to exhibit critical roles in the development of gastric cancer [118]. gp130 is the signaling component of the IL-6 receptor complex, which can interact with tyrosine phosphatase Shp2 and STAT3 by differential targeting of specific phosphotyrosine residues in the intracellular tail of gp130. Genetic manipulation of the gp130 receptor, which modulates the balance of the Shp2/ERK and JAK/STAT signal transduction pathways, enhanced peptic ulceration and gastric cancer in transgenic mice [119]. The host cell factors that are targeted upon *H. pylori* infection include the Shp2/ERK or JAK/STAT3 cascades, implicating that *H. pylori*-associated pathogenicity could be influenced by gp130 signaling [94,120,121]. In fact, the role of translocated CagA in the activation of Shp2/ERK and JAK/STAT downstream of gp130 has been investigated [122]. It was shown that CagA^PY^ recruited Shp2 to gp130, which stimulated ERK1/2 MAP kinase phosphorylation (Figure 1F). Interestingly, while CagA^PY^ enhanced Shp2 binding capacity, the expression of phospho-deficient CagA predominantly activated STAT3 [122]. Known targets of the pathway include antibacterial REG3γ, a C-type lectin, exerting direct bactericidal activity against Gram-positive bacteria [123]. This response might allow Gram-negative *H. pylori* to eliminate competing Gram-positive bacteria in the gastric niche. Taken together, the above findings indicate that the phosphorylation status of translocated CagA determines a signaling switch between the Shp2/ERK and JAK/STAT3 pathways through gp130, providing an elegant new mechanism showing how *H. pylori* hijacks disease-related signaling cascades in epithelial cells, affecting bacterial survival and gastric pathogenesis.

## 12. Summary

*H. pylori* represents a highly successful human pathogen, which can trigger severe clinical diseases in a subset of patients. The investigation of bacteria-host interactions and virulence factors such as the T4SS and CagA have provided us with crucial insights into the mechanisms leading to *H. pylori* pathogenesis. Research in recent years has generated considerable progress in our understanding of the structural biology and function of CagA. Various reported CagA structures, together with biochemical and functional analyses have revealed key parameters to enable multifaceted interaction complexes. A list of more than 25 known cellular binding partners of CagA is quite amazing for a bacterial effector protein. Among them are at least seven host cell receptor molecules in the gastric epithelium, whose action has been reviewed in the present article. The current model suggests that CagA mimics a eukaryotic signaling factor either located in a large multiprotein complex or simultaneously in various subcellular areas of infected host cells. The large variety of binding partners also reflects the integrated network of signal transduction pathways in target cells, which may have an important impact on the multi-step pathogenesis of *H. pylori*. However, there are still many fundamental questions regarding the translocation of CagA into the host cell as well as signaling that need to be addressed in future. For example, while we accumulated substantial knowledge in recent years on CagA internalization and signaling in gastric epithelial cells, very little is known about the interaction of the T4SS with immune cells. CagA appears to be rapidly translocated and phosphorylated in various immune cell types, but undergoes cleavage into 100-kDa N-terminal and 35-kDa C-terminal fragments, whose functions are not yet known [124,125,126]. Various crystal structures of CagA and CagA in complex with cellular partners are available and have proven to be extremely informative. One remarkable outcome of the discoveries made during the past few years is that while N-terminal CagA and C-terminal CagA have obviously different structural properties; they both contain important, dedicated interaction modules. In the future, structural information on the *cag* T4SS and full-length CagA as well as of the numerous complexes they form with host cell proteins should provide more detailed and mechanistic insights. In turn, this information might be translated into novel strategies to develop small inhibitor compounds, which could inhibit this sophisticated T4SS and help to eradicate *H. pylori* or combat gastric diseases.

## Abbreviations

aa (amino acid), Akt (serine/threonine kinase; also called protein kinase B, PKB), Abl (Abelson kinase), *cag* (cytotoxin-associated genes), Cbl (Casitas B–lineage lymphoma protein), CEACAM (Carcinoembryonic antigen-related cell adhesion molecule), CSP (Conserved surface-exposed patch), EGF (Epidermal growth factor), EGFR (Epidermal growth factor receptor), ERK (Extracellular regulated kinase), FAK (Focal adhesion kinase), F-BAR (Fes-CIP4 homology-Bin/Amphiphysin/Rvs), Gab-1 (Grb2-associated binder 1), GGT (γ-glutamyl- transpeptidase), Grb-2 (Growth factor receptor-bound protein 2), GSK-3β (Glycogen synthase kinase 3beta), gp130 (glycoprotein 130), hBD3 (human beta-defensin 3), HGF (Hepatocyte growth factor), Jak (Janus kinase), MALT (Mucosa-associated lymphoid tissue), MCL1 (Myeloid leukemia cell differentiation protein 1), MEK (Mitogen-activated protein kinase kinase), MMP (Matrix metalloproteinase), NF-κB (Nuclear factor kappa B), OMV (outer membrane vesicle), p120^ctn^ (p120-catenin), PAI (Pathogenicity island), PH (Pleckstrin homology), PLCγ (phospholipase C-γ), Raf (Rapidly accelerated fibrosarcoma kinase), Ras (Rat sarcoma GTPase), RTK (Receptor tyrosine kinase), Shp2 (Src homology 2-containing phosphotyrosine phosphatase), Src (Sarcoma kinase), STAT-3 (Signal transducers and activators of transcription-3), T4SS (Type IV secretion system), TCF/LEF (T-cell factor/Lymphoid enhancer factor), TF (Transcription factor), T4SS (Type IV secretion system), VacA (Vacuolating cytotoxin A) and GGT.
